# Rockburst grade evaluation and parameter sensitivity analysis based on SSA-PNN framework: Considering rock mass strength

**DOI:** 10.1371/journal.pone.0325966

**Published:** 2025-06-24

**Authors:** Xiaoyan Zhou, Yimin Jiang, Zhenyi Wang, Yalei Wang

**Affiliations:** 1 School of Architectural Engineering, Zhengzhou University of Industrial Technology, Zhengzhou, China; 2 Department of Civil Engineering, Shanghai University, Shanghai, China; China Construction Fourth Engineering Division Corp. Ltd, CHINA

## Abstract

The occurrence of rockburst is closely related to the strength and stress conditions of rock mass. The Lalin Railway tunnel in China was taken as an example, the strength and stress parameters of rock mass at 22 rockburst locations were obtained by using the results of indoor and outdoor tests, including maximum in-situ stress, maximum tangential stress, uniaxial compressive strength of rock and uniaxial compressive strength of rock mass. These four parameters were then selected to form a rockburst grade evaluation index system. Furthermore, SSA (Sparrow search algorithm) and probabilistic neural network (PNN) were used to construct a rockburst grade evaluation network, and the sensitivity of rockburst grade evaluation parameters was therefore analyzed. It shows that SSA could determine the smoothness factor of PNN efficiently, and it is reasonable to use SSA-PNN framework to evaluate the rockburst grade; maximum tangential stress and uniaxial compressive strength of rock mass have the greatest influence on the accuracy of rockburst grade evaluation, followed by maximum in-situ stress, and uniaxial compressive strength of rock has the least influence on the accuracy of rockburst grade evaluation; integrated maximum in-situ stress, maximum tangential stress, uniaxial compressive strength of rock and uniaxial compressive strength of rock mass, the rockburst grade evaluation results are highly reliable. The results presented herein may provide important reference value for the rockburst grade evaluation and the selection of rockburst grade evaluation parameters.

## 1. Introduction

Rockburst is a violent phenomenon caused by the sudden release of internal stress in rock mass, which is often accompanied by serious disasters such as rupture, vibration and flying rocks, and poses a threat to the safety of mining, tunnel construction and underground engineering. With the increase of mining depth and the complexity of underground engineering, the occurrence mechanism and prediction of rockburst become more and more complicated [1–6]. Therefore, the evaluation of rockburst grade and the sensitivity of its influencing factors is one of the hot spots in the current geological engineering research.

In recent years, rockburst grade evaluation methods based on rock strength have made remarkable progress in the understanding of rockburst mechanism, the improvement of the accuracy of evaluation results and the development of numerical simulation techniques. For example, Liu et al. [7] established a new criterion of stress-intensity ratio rockburst considering the stress gradient of surrounding rock based on the statistics of a large number of engineering rockburst cases; Khiadivi et al. [8] discussed the relationship between rockburst and rock brittleness, and then evaluated the grade of rockburst based on the brittleness of rock; Li et al. [9] proposed a new method for rockburst grade prediction based on rock strength, stress conditions and elastic energy index; Sun et al. [10] carried out rockburst simulation tests of heterogeneous granite under true triaxial single-side unloading conditions, and analyzed rockburst ejection damage characteristics, strength and deformation characteristics, acoustic emission characteristics, rock fragment characteristics and kinetic energy evolution laws of rock samples; Yan et al. [11] analyzed the failure mechanism of rock under the coupled action of static and dynamic loads and the influence of rock strength on rockburst; Chen et al. [12] explored the influence of the size effect of uniaxial compressive strength and deformation modulus ratio on the rockburst occurrence process; Wu et al. [13] explored the influence of vertical prestress and dynamic load on the occurrence of rockburst under high stress conditions; Li et al. [14] proposed a macro-microscopic mechanical model to describe the above rock physical and mechanical properties, and combined the obtained model with the brittleness evaluation index and the residual elastic energy index, respectively, to analyze the influence of microscopic parameters and principal stress on rockburst tendency. Researches on rockburst grade evaluation based on rock strength have certain application value, but rock strength is usually a single physical property index, which fails to fully consider the influence of weathering degree of rock mass, fracture development degree and other factors, which makes the research on rockburst lack a comprehensive reflection of the actual complex geological conditions.,

In fact, the occurrence of rockburst is not only related to the mechanical properties of rock (such as strength, hardness, etc.), but also closely related to the structural characteristics of rock mass. The rock mass heterogeneity, fracture system, joint plane, bedding structure and other factors play a decisive role in the occurrence of rockburst [15]. Recently, rockburst research based on the structural characteristics of rock mass has gradually become an important research direction. For example, Cai et al. [16] studied the influence of water injection on the surface of tunnel rock mass on the internal mechanism of rockburst occurrence; Pan et al. [17] discussed the influence of joint density on the occurrence process of rockburst based on the numerical simulation test of rockburst; Zhu et al. [18] proposed a multi-factor rockburst grade evaluation method that comprehensively considered rock properties, rock integrity and elastic strain energy released by surrounding rock; He et al. [19] studied the influence of layered joints with different dip angles and spacing on rockburst in deep buried tunnels; Wu et al. [20] studied the influence of different rock mass structures on the rockburst occurrence process during tunnel excavation through rockburst model and numerical simulation test; He et al. [21] studied the influence of different ground stress values and joint angles on rockburst in deep buried tunnels under strong dynamic disturbance; Yao et al. [22] studied the influence of structural parameters of rock mass on the occurrence of rockburst under different stress paths; Chen et al. [23] studied the fracture propagation behavior in jointed rock mass and evaluated the influence of borehole pressure relief on reducing rockburst occurrence; Dai et al. [24] investigated the influence of excavation weakening effect of rock mass parameters on rockburst occurrence; Qi et al. [25] used the revised rock mass integrity coefficient and geological strength index to predict the rockburst grade. The above studies have considered the effects of fractures, joints, stresses and geological structures on the occurrence of rockburst, and achieved a lot of research results, but most of the research results focus on the analysis of rock mass structure on rockburst mechanism, and few studies on the evaluation of rockburst grade. Moreover, most of the methods used for rockburst grade evaluation are single index methods based on rock strength and stress conditions, but the evaluation information of single index evaluation method is relatively simple.

At the same time, considering the complexity of rockburst itself, intelligent evaluation methods based on machine learning and data mining have gradually become a new way to solve the difficult problem of rockburst grade evaluation [26,27]. Wu et al. [28] constructed a rockburst grade evaluation model using principal component analysis method and probabilistic neural network; Chen et al. [29] evaluated the rockburst grade using the Gaussian Naive Bayes method; Li et al. [30] established a rockburst grade evaluation method based on an improved feed-forward neural network using the Bayesian optimization method; Shen et al. [31] aiming at the limitations of traditional machine learning models in explaining the prediction process of rockburst intensity, proposed a rockburst grade evaluation model based on Optuna-random forest; Xiao et al. [32] proposed a rockburst grade evaluation network based on Kernel Extreme Learning Machine combined with Genetic Algorithm and cross-entropy method. The rockburst grade evaluation neural network method reveals the rockburst mechanism from different angles and enriches the rockburst rating evaluation method. However, the rockburst rating evaluation method mainly starts from the ground stress and rock mechanics characteristics, and ignores the influence of rock structure characteristics on rockburst. Therefore, the research on the sensitivity of influencing factors of rockburst is still weak. How to combine different intelligent algorithms to optimize the comprehensive effect of rockburst grade evaluation is an important research direction. Furthermore, in the traditional neural network for evaluating rockburst grades, the selection of parameters often relies on human experience and requires a large amount of human trial-and-error time, which is not conducive to the improvement of network accuracy and generalization ability. Therefore, it is necessary to add the rockburst grade evaluation index to the neural network evaluation method, analyze the sensitivity of the evaluation index to the rockburst grade evaluation results, and establish an accurate and efficient intelligent rockburst grade evaluation network.

Accordingly, taking Lalin Railway tunnel project as an example, the in-situ stresses of 22 rockburst areas were obtained by using hollow inclusion stress relief method, and the maximum tangential stress of surrounding rock tunnel wall was then obtained through translation. The uniaxial compressive strength of rock mass was estimated according to the structural characteristics of rock mass in the rockburst area, and the uniaxial compressive strength of rock mass was also obtained through laboratory tests. The maximum in-situ stress, maximum tangential stress of tunnel, uniaxial compressive strength of rock and uniaxial compressive strength of rock mass were then selected to form the evaluation index system of rockburst grade.

The SSA (sparrow search algorithm, SSA) is an intelligent optimization algorithm proposed in 2020 to simulate the foraging and anti-predation behaviors of sparrow populations [33]. This algorithm has the characteristics of strong optimization ability and fast convergence speed. The PNN (Probabilistic Neural Network, PNN) was proposed in 1990 [34]. It has the advantages of simple structure, easy training, fast convergence speed, strong fault tolerance ability, and can implement the functions of nonlinear learning algorithms using linear learning algorithms. The combination of SSA and PNN can avoid reliance on human experience and solve problems such as traditional models being prone to fall into local optima, slow training, and poor performance with small samples. Therefore, SSA and PNN were used to construct a rockburst grade evaluation network, and the sensitivity analysis of the key parameters affecting the evaluation results of the SSA-PNN framework was also carried out.

## 2. Engineering overview of the Lalin Railway Tunnels

Lalin Railway is an important railway project connecting Lasha and Linzhi in Tibet, China, with a total length of about 435 kilometers, of which the tunnel section accounts for about 60% of the total length. This railway passes through the Himalayan mountains and plateau areas, and the complex geological conditions, especially the problem of rockburst, pose a major challenge to the safety and schedule of the project during tunnel construction. The geological background of the tunnel is complex, covering a variety of rock formations such as metamorphic rock, granite, gneiss, etc., especially some deep tunnel sections, such as Bayu Tunnel and Sangzhuling Tunnel, are located in high-stress rock formations and have a high frequency of rockbursts.

### 2.1. Geological conditions

The geological section through which Lalin Railway tunnel passes is dominated by plateau crust and its overlying metamorphic rock series, with complex geological conditions and surrounding rock generally in a state of high stress and high pressure. In the process of tunnel construction, large scale fault, fold and rock fracture zone are often encountered, especially in the area with large tunnel excavation depth, the stress of surrounding rock is concentrated, and rockburst occurs frequently. Specifically, the risk of rockburst is high in some tunnel sections, especially in Sangzhuling Tunnel, where the surrounding rock pressure reaches 30–50 MPa and the local rock stress exceeds 70 MPa, resulting in rockburst events. According to the preliminary geological survey, the tunnel section of Lalin Railway is mostly located in the strong metamorphic and hard rock strata, the stress level of deep rock strata is high, and the crack development of rock mass itself makes the risk of rockburst increase in tunnel construction.

### 2.2. Rockburst problem

**Fig 1:**
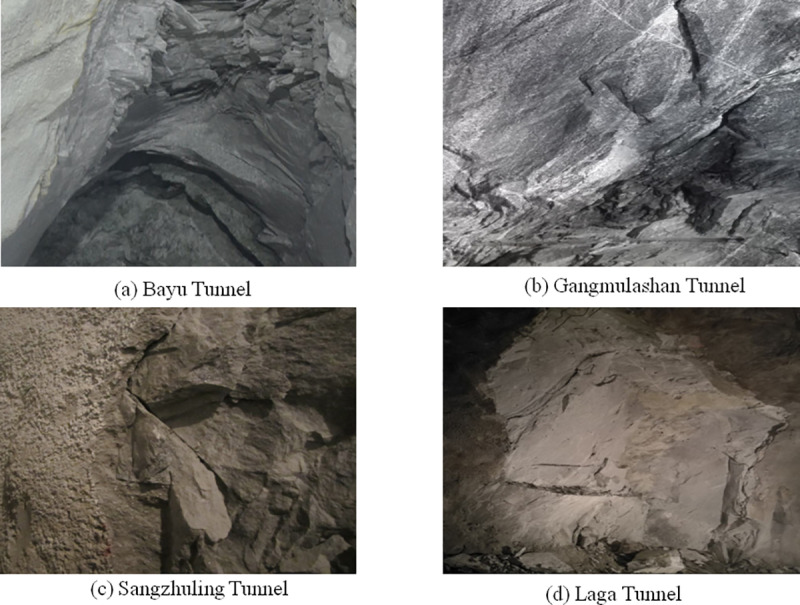
Appearance of different tunnel rockburst locations.

The rockburst phenomenon of Lalin railway tunnel mainly occurs in rock strata with hard lithology and high stress, such as granite and diorite. Rockburst not only affects the construction safety, but also may cause equipment damage and increase the difficulty of construction. In key sections such as Bayu Tunnel, Gangmulashan Tunnel, Sangzhuling Tunnel and Laga Tunnel (see Fig 1), the frequency of rockburst is relatively high. According to the analysis of engineering data, several strong rockburst events occurred in some sections, and the maximum explosive intensity was strong. In order to effectively deal with this problem, the construction unit has taken measures such as advanced geological exploration, reinforcement of supporting structure (such as steel arch and shotcrete) and optimization of excavation scheme to reduce the risk of rockburst.

## 3. Rockburst influence parameters

Rockburst is a complex geological disaster. The causes that induce rockburst are mainly divided into internal and external factors. The internal factor is mainly lithological conditions, while the external factor is mainly stress conditions. Therefore, in this study, both the internal and external causes that induce rockburst were comprehensively considered. Combined with the representativeness principle, quantitative analysis principle and operability principle of characteristic indicators, the maximum tangential stress and maximum in-situ stress of the surrounding rock, the uniaxial compressive strength of the rock and the uniaxial compressive strength of the rock mass were selected as the inputs of the network. Among them, the uniaxial compressive strength of rocks and the uniaxial compressive strength of rock masses reflect the lithological conditions, while the maximum in-situ stress of rock masses and the maximum tangential stress of tunnel represent the stress conditions. The following is a brief introduction to obtain the maximum in-situ stress, maximum tangential stress of tunnel, uniaxial compressive strength of rock mass and uniaxial compressive strength of rock mass.

### 3.1. Determination of stress condition of rock mass

**Fig 2:**
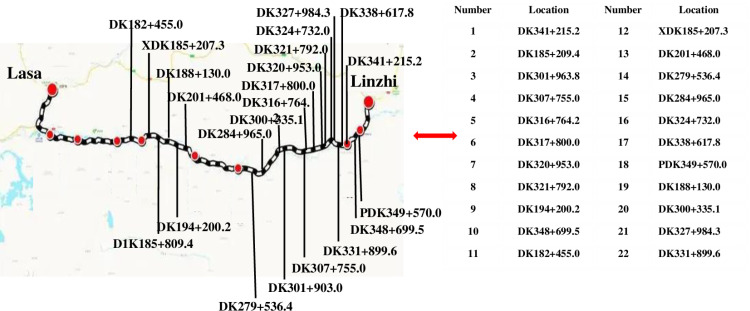
Measuring point arrangement of in-situ stress. The testing process of in-situ stress was shown in Fig 3.

In order to obtain the stress condition of rock mass at different rockburst locations, the in-situ stress of rock mass at 22 rockburst locations in Lalin railway tunnel was measured by using hollow core inclusion stress relief method. The arrangement of measuring points is shown in Fig 2.

**Fig 3:**
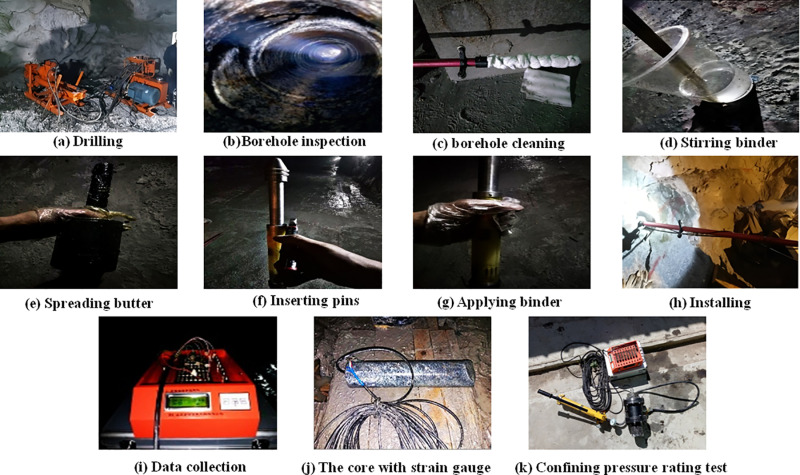
The testing process of in-situ stress.


According to the steps in Fig 3, the in-situ stress test results of rockburst locations of different grades can be obtained, and the in-situ stress test results are converted through formula (1) ~ (4) to obtain the maximum tangential stress of the tunnel at 22 in-situ stress measurement points where rockburst occurred in Lalin Railway tunnel. The results are shown in Table 1.

**Table 1:** Maximum tangential stresses of tunnels at different rockburst locations.

Number	Drilling azimuth/ °	*α* / °	*σ*_max_ /MPa	*σ*_Hmax_ /MPa	*σ*_Hmin_ /MPa	*σ*_V_ /MPa	*σ*_θmax_ /MPa
1	86.7	87.2	49.7	31.0	18.5	9.2	66.0
2	99.5	0.6	23.5	23.5	14.9	14.4	56.1
3	145.5	80.8	22.2	22.2	13.7	10.6	45.3
4	67.1	18.7	34.8	34.8	14.7	19.6	83.2
5	150.2	37.2	19.9	19.9	10.4	12.3	44.5
6	101.5	34.2	43.3	43.3	17.8	20.4	102.9
7	120.1	49.7	31.8	31.8	14.2	17.2	68.9
8	81.3	37.7	28.6	28.6	7.9	14.4	64.9
9	7.1	77.4	31.0	49.70	36.10	37.0	96.2
10	30.1	84.6	33.3	33.3	16.2	18.9	57.8
11	4.5	89.8	45.4	45.4	20.8	25.4	74.0
12	37.6	53.7	36.6	36.6	14.0	19.9	76.1
13	11.0	87.7	48.6	48.7	30.0	25.1	96.0
14	148.1	44.1	28.8	28.8	13.2	14	65.8
15	161.2	30.1	29.6	29.6	15.2	15	70.9
16	177.3	86.2	28.7	28.7	17.0	20.7	49.0
17	17.2	83.5	24.9	24.9	4.7	7	40.8
18	4.8	67.5	35.8	35.8	12.8	26.5	59.6
19	10.2	52.4	78.7	78.7	24.5	33.8	170.6
20	142.6	89.7	25.4	25.4	12.0	13.3	42.9
21	15.1	77.7	36.9	36.9	19.6	30.1	60.2
22	20.8	80.9	46.3	46.3	21.2	15.5	91.7

$$\sigma_{D}\;=\;0\mathrm{.5}\;\times \;(\sigma_{Hmax}\;+\;\sigma_{Hmin})\;+\;0\mathrm{.5}\;\times \;(\sigma_{Hmax}\;-\;\sigma_{Hmin})\;\times \;cos2\alpha$$ (1)

$$\sigma_{L}\;=\;0\mathrm{.5}\;\times \;(\sigma_{Hmax}\;+\;\sigma_{Hmin})\;-\;0\mathrm{.5}\;\times \;(\sigma_{Hmax}\;-\;\sigma_{Hmin})\;\times \;cos2\alpha$$ (2)

$$\sigma_{\theta max}\;=\;3\sigma_{D}\;-\sigma_{V},\;\;\sigma_{D}\;\geq \;\sigma_{V}$$ (3)

$$\sigma_{\theta max}\;=\;3\sigma_{V}-\sigma_{D},\;\sigma_{D}<\sigma_{V}$$ (4)

Where, *σ*_D_ represents the horizontal stress perpendicular to the tunnel axis; *σ*_L_ represents the horizontal stress parallel to the tunnel axis; *α* represents the angle between the direction of maximum horizontal principal stress and the direction of tunnel; *σ*_θmax_ represents the maximum tangential stress of the tunnel; *σ*_Hmax_ means maximum horizontal principal stress; *σ*_Hmin_ means minimum horizontal principal stress; *σ*_V_ stands for vertical principal stress.

### 3.2. Determination of rock mass strength

Rock mass is usually composed of rock blocks and weak structural planes. Although the in-situ stress of rock mass can be measured by stress relief method, the uniaxial compressive strength of rock mass cannot be directly measured by test [35]. Moreover, the interaction between rock block and joint fracture is complicated, so it is difficult to determine the uniaxial compressive strength of rock mass according to the test results of small size rock. Although various field tests, such as block shear, plate load or slab jacking test, can provide some relevant information about rock mass strength, the cost of field tests is often high and the actual operation is difficult. It is not a good choice to determine the uniaxial compressive strength of rock mass through a large number of field test methods in deep buried tunnel engineering. The uniaxial compressive strength of rock mass is estimated by some empirical methods. At present, there are many methods to estimate uniaxial compressive strength of rock mass, but the generalized Hoek-Brown (H-B) strength criterion is the most widely used. The generalized H-B strength criterion suggests that formula (5) ~ (8) be used to estimate the uniaxial compressive strength of rock mass.


$$\sigma_{cm}\;=\;\sigma_{Ci}\;S^{a}$$ (5)

$$m\;=\;m_{i}e^{\left(GSI-100)/(28-14D\right)}$$ (6)

$$a\;=\;0.5\;+\;(e^{-GSI/15}-e^{-20/3})/6$$ (7)

$$s\;=\;e^{(GSI-100)/(9-3D)}$$ (8)

**Fig 4:**
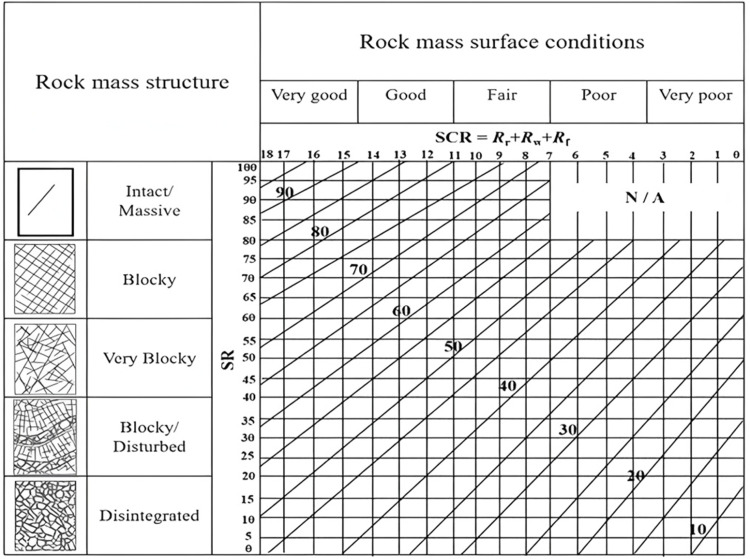
GSI value method [36].

Where, *σ*_cm_ is the uniaxial compressive strength of rock mass, and the unit is MPa; *σ*_ci_ is the uniaxial compressive strength of rock, expressed in MPa, which is obtained by laboratory uniaxial compression test; the empirical parameter *m* of rock mass is the *m*_i_ discount value considering rock mass structure; *m*_i_ is an empirical parameter that reflects the softness and hardness of a rock; GSI is an indicator of geological strength, and its value method is shown in Fig 4.

**Fig 5:**
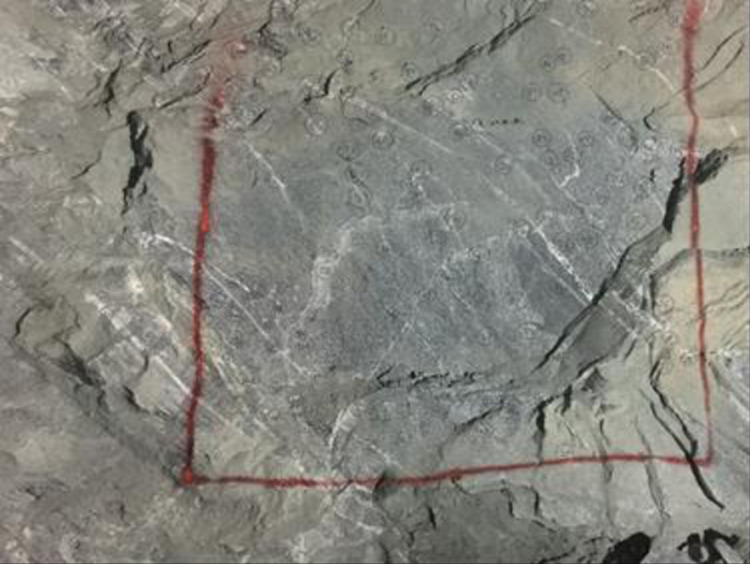
Structure plane of rock mass at measuring point 1.

Taking the in-situ stress measuring point 1 as an example, the estimating process of uniaxial compressive strength of rock mass was briefly explained. In order to estimate the structural parameters of the rock mass at this location, the area of 2 m × 2 m (see Fig 5) was measured.

**Fig 6:**
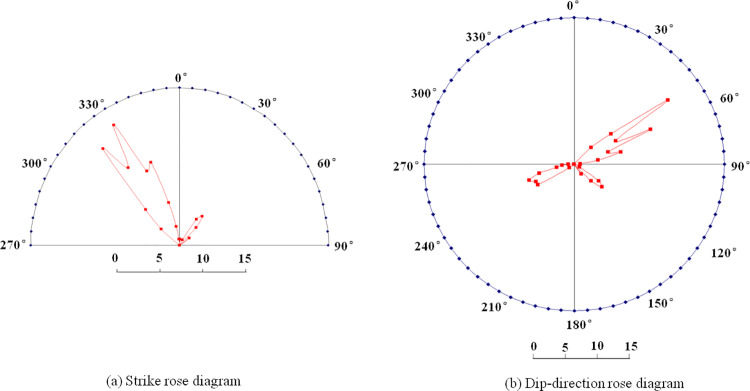
Rose diagram of joints.

As shown in Fig 5, the structural surface of the rock mass at this location is very rough and unweathered, and the cracks in the structural surface are full of fillers (mostly calcite) with an opening degree of 5 ~ 37 mm. Accordingly, the surface condition rating (SCR) of rock mass structure is estimated to be 14. The joints in this area are relatively developed and distributed in an “X” shape. Joints generally do not extend long. In the 126 groups of joint occurrence test results, the joint strike was converted into NE (0° ~ 90°) and NW (270° ~ 360°) directions, one group was set every 5°, the number of joints in each group was counted, the average strike and average tendency of joints in each group were calculated, and the distribution characteristics of joint occurrence were analyzed using the joint rose diagram, as shown in Fig 6.

As can be seen from Fig 6, the dominant orientation of the joint can be divided into four groups according to dip-direction: The first group has a dip-direction of 45° ~ 60°, accounting for 31.0% of the total number of joints, and an average tendency of 54.0°; The second group has a dip-direction of 65° ~ 80°, accounting for 25.4% of the total joints and an average dip-direction of 70.3°. The third group has a dip-direction of 120° ~ 135°, accounting for 14.3% of the total joints and an average tendency of 125.9°. The fourth group has a tendency of 240° ~ 255°, accounting for 24.6% of the total number of joints, and an average dip-direction of 244.8°. According to the average spacing of each group of joints, it is estimated that the number of joints in rock mass is about 15.5. Accordingly, structure rating (SR) was estimated to be 31.8 (the integer 32 is used to facilitate the calculation of SR). By substituting SCR (14) and SR (32) into the GSI value table, the GSI is about 51. The tunnel excavation adopts drilling and blasting, and the D of the rock mass at the construction site is 0.5. The lithology of the rock is granite *m*_i_ 33, and the parameters m and s of the rock mass are 3.2001 and 0.001454, respectively, when GSI, *m*_i_ and D are substituted into the formula (6) ~ (8). According to formula (5), the rock mass strength at measuring point 1 is 3.72 MPa.

Similarly, the uniaxial compressive strength of rock mass at the remaining measuring point can be obtained, and the uniaxial compressive strength of rock can be determined by the indoor uniaxial compression test. For the convenience of analysis, the table also lists the maximum in-situ stress, the maximum shear stress, the uniaxial compressive strength of the rock and the rockburst grade of the rock mass at the measuring point. The results were shown in the Table 2.

**Table 2:** Uniaxial compressive strength of the rock mass.

Number	grades	*σ*_θmax_ /MPa	*σ*_max_ /MPa	*σ*_ci_ /MPa	*σ*_cm_/MPa	Number	grades	*σ*_θmax_ /MPa	*σ*_max_ /MPa	*σ*_ci_ /MPa	*σ*_cm_ /MPa
1	Medium	66.0	49.7	101.27	3.72	12	Strong	76.1	36.6	43.15	2.09
2	Medium	56.1	23.5	35.63	2.12	13	Medium	96.0	48.6	123.03	8.42
3	Slight	45.3	22.2	84.54	4.10	14	Medium	65.8	28.8	78.87	3.57
4	Strong	83.2	34.8	30.43	1.47	15	Medium	70.9	29.6	88.76	4.31
5	Medium	44.5	19.9	22.15	1.51	16	Medium	49.0	28.7	41.10	2.13
6	Strong	102.9	43.3	21.08	1.02	17	Medium	40.8	24.9	74.83	3.16
7	Strong	68.9	31.8	26.51	1.20	18	Medium	59.6	35.8	48.40	2.69
8	Medium	64.9	28.6	43.49	2.11	19	Strong	170.6	78.7	63.97	3.32
9	Strong	96.2	31.0	40.62	2.42	20	Medium	42.9	25.4	50.37	2.80
10	Medium	57.8	33.3	62.88	2.66	21	Medium	60.2	36.9	55.39	3.08
11	Medium	74.0	45.4	100.97	5.25	22	Strong	91.7	46.3	42.54	2.21

The rockburst grades of the rock mass at the measurement points are also listed in Table 2. The actual rockburst grade is mainly determined comprehensively through characteristics such as sound, movement, aging, influence depth and hazardous degree. The specific classification method of rockburst grades is shown in Table 3.

**Table 3:** The classification of rock burst grade [37].

Features of rock burst	Rock burst grades			
no	slight	medium	strong
Sound features	None	Cracking sounds and tearing sounds	The crisp crackling sound	Very loud popping sounds
Movement features	None	loosen or peel off	Burst, peeled off, and little ejection	Lots of bursts and ejections
Aging features	None	Occurs sporadically and intermittently	Long duration	Occurs in succession and rapidly extends deep
Influence depth	None	<0.5m	0.5 to 1 m	1 to 3 m
hazardous degree	None	Little	Relatively large	Serious

## 4. Rockburst grade evaluation network based on SSA-PNN framework

Compared with traditional methods, SSA (sparrow search algorithm)-PNN (probabilistic neural network) could avoid reliance on human experience and solve problems such as traditional models being prone to fall into local optima, slow training, and poor performance with small samples. Probabilistic output is more in line with the requirements of engineering risk assessment than a single classification label, providing a quantitative basis for prevention and control measures. Through the collaborative optimization of SSA and PNN, efficient, high-precision and strongly robust modeling can be achieved in the evaluation of rockburst grades, providing reliable technical support for the safety of underground engineering. Therefore, the rockburst grade evaluation network based on SSA and PNN is an integrated optimization and machine learning model for effectively evaluating rockburst grades. Rockburst is usually caused by underground mining and deep mine work under the stress change of rock mass sudden rupture or eruption phenomenon, causing great harm to mines and tunnels and other projects, so accurate evaluation of rockburst occurrence grade is very important for safety assessment.

### 4.1. Sparrow search algorithm

SSA is a swarm intelligent optimization algorithm that simulates the foraging behavior of sparrows. Inspired by sparrow’s foraging behavior, this algorithm simulates the cooperative and competitive mechanism of sparrow in the process of finding food. SSA combines swarm intelligence, local search and global search, and can effectively solve various optimization problems. The main steps of the sparrow search algorithm are as follows:

(1) Initialization. The position and speed of a group of sparrows (i.e., multiple candidate solutions in the solution space) were initialized. According to the dimensions and constraints of the problem, the position of each sparrow was then randomly generated.
(2) Fitness evaluation. The fitness calculation is performed for the current position of each sparrow (the fitness function usually depends on the specific optimization problem, and the goal is to minimize or maximize some function value).
(3) Update sparrow position. According to different types of sparrows, there are two types of updates: Some good sparrows act as leaders, and the leader looks for the best solution and guides other sparrows; other sparrows move in the direction of the leader and may conduct local exploration.
(4) Fitness selection. After each iteration, the sparrow with the best fitness is selected as the current leader, and the position is updated according to its guidance to other sparrows.
(5) Iterative process: Continue iterations, with each iteration updating the sparrow’s colony position until termination conditions are met (such as reaching a maximum number of iterations or finding a satisfactory solution).

For the leader Sparrow, its position update can be done by the following formula:


$$X_{i}(t+1)\;=\;X_{i}(t)\;+\;\gamma \;(X_{best}\;(t)-X_{i}(t))+a\;(X_{i}(t)-X_{r})$$ (9)

Where, *X*_i_(t) is the position of the leader sparrow in the *t* iteration; *X*_best_ (*t*) is the optimal position for the number of iterations *t*; *X*_r_ is a randomly selected sparrow position; *a* and *γ* are the parameters of regulation.

For the follower sparrow, the position update formula is usually based on the position information of the finder sparrow:


$$X_{j}(t+1)\;=\;X_{j}(t)+\beta \;(X_{i}(t)-X_{j}(t))$$ (10)

Where, *X*_j_(*t*) is the position of the follower sparrow in the *t* iteration; *β* is the follower’s learning factor.

### 4.2. Probabilistic neural network

PNN is a feedforward neural network based on Bayesian theory, which is mainly used for classification problems, especially for pattern recognition tasks. Unlike traditional multi-layer perceptrons or other neural networks, PNN classifies by estimating the probability distribution of the data, and therefore shows better performance in some classification tasks, especially in cases where the data sets are small. The core idea of PNN is to use Bayesian decision theory to estimate the probability density around the sample points of each class by kernel functions (such as Gaussian kernels), and then classify according to these estimates.

The structure of PNN. PNN network is mainly composed of the following four layers: input layer, pattern layer, summation layer and output layer.

Input layer: the input layer directly accepts feature vectors, one for each node; each node of the input layer passes the characteristics of the input to the next layer.

Pattern layer: the number of nodes in the pattern layer is equal to the number of training samples; each node represents a training sample, and similarity is assessed by calculating the distance between that sample and the input data; typically, a Gaussian kernel function is used to calculate similarity.


$$K\left(x,x_{i}\right)=exp(-\frac{{\left\|x-x_{i}\right\|}^{2}}{2\sigma^{2}})$$ (11)

Where, *x* is the input sample; *x*_i_ is the training sample; *σ* is the width parameter of the Gaussian kernel, which usually needs to be adjusted during training.

Summation layer: The summation layer is responsible for summarizing the probability density for each category; each category corresponds to a summary node, and each node in the summary layer adds the outputs of all nodes belonging to that category in the pattern layer to get the overall probability density for that category.

Output layer: the output layer is responsible for converting the results of the summary layer into the final classification results; for each category, the output layer selects the category with the highest probability density as the final prediction result.

### 4.3. Establishment of rockburst grade evaluation network based on SSA-PNN

**Fig 7:**
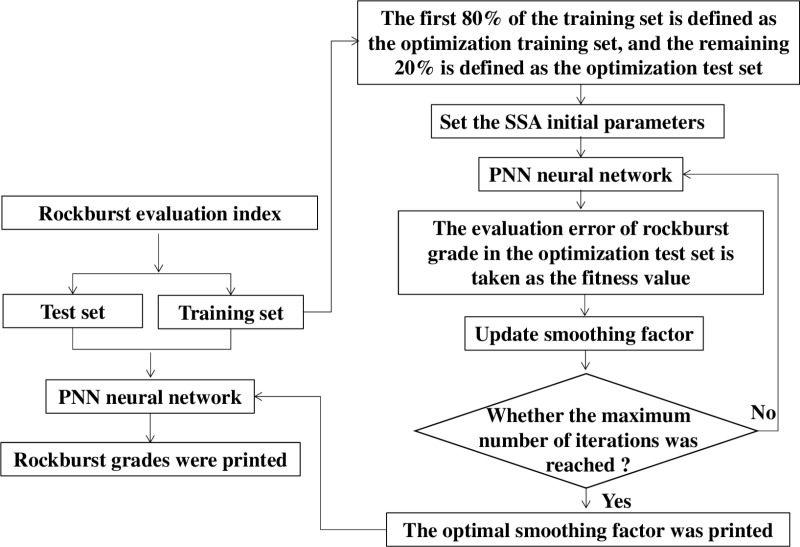
The structure of SSA-PNN framework.

The occurrence mechanism of rockburst is complicated and affected by many factors such as geological structure and stress conditions, but it can be generally summarized as the internal conditions dominated by the underlying lithology and the external conditions dominated by the stress state of rock mass. Randomly select 12 samples in Table 2 as the training set, and the remaining 10 samples as the test set. In order to avoid abnormal and noisy data, speed up network training and improve network training accuracy, the initial data is standardized, and the standardized data is input into the PNN network. Fig 7 shows the structure of the multi-index rockburst grades evaluation method SSA-PNN.

The performance of PNN significantly depends on the selection of the smoothing factor *σ*. Usually, the smoothing factor *σ* is selected as a fixed value based on experience, but this lacks a solid theoretical basis in engineering practice, resulting in its limited application. In this study, smooth factor *σ* is a key parameter to evaluate the performance of PNN networks: too small smooth factor will easily cause the network to over-fit, while too large smoothness factor tends to make the network unable to distinguish details. Moreover, in order to improve the generalization ability of PNN and quickly find the optimal smoothing factor, the first 80% of the training set was defined as the optimization training set, and the remaining 20% was defined as the optimization test set. Then the divided training set was input into SSA and PNN. In this study, based on the training set data, the SSA algorithm was used to optimize the PNN network, and the optimal smoothness factor (0.01) was obtained. Accordingly, a multi-index rockburst grade evaluation network based on SSA-PNN framework was constructed.

### 4.4. Evaluation results of rockburst grade evaluation network based on SSA-PNN

The SSA-PNN framework was used to evaluate the rockburst grade of the test set, and the results were shown in Table 4. As can be seen from Table 4, the evaluation results of SSA-PNN framework are basically consistent with the actual rockburst grade, and the accuracy rate is about 90%; The SSA-PNN framework may over-evaluate the slight rockburst.

**Table 4:** Rockburst grade evaluation results based on SSA-PNN framework.

Number	*σ*_θmax_ /MPa	*σ*_max_ /MPa	*σ*_ci_ /MPa	*σ*_cm_ /MPa	Actual rockburst grade	Evaluate results
1	96.2	31.0	40.62	2.42	IV	IV
2	56.1	23.5	35.63	2.12	III	III
3	45.3	22.2	84.54	4.10	II	III
4	83.2	34.8	30.43	1.47	IV	IV
5	44.5	19.9	22.15	1.51	III	III
6	102.9	43.3	21.08	1.02	IV	IV
7	68.9	31.8	26.51	1.20	IV	IV
8	64.9	28.6	43.49	2.11	III	III
9	66.0	49.7	101.27	3.72	III	III
10	57.8	33.3	62.88	2.66	III	III

In order to analyze the application of different optimization calculations in PNN, the rockburst grades of the validation set were evaluated using the PNN, SSA-PNN, GA-PNN and PSO-PNN frameworks respectively, and the results are shown in the Table 5.

**Table 5:** Evaluation results various rock burst evaluation methods.

Number	Various rock burst evaluation methods				Actual rockburst grade
PNN	SSA-PNN	PSO-PNN	GA-PNN
1	III	IV	IV	IV	IV
2	IV	III	III	III	III
3	III	III	III	III	II
4	IV	IV	IV	IV	IV
5	IV	III	III	III	III
6	IV	IV	IV	IV	IV
7	IV	IV	III	III	IV
8	III	III	III	III	III
9	III	III	III	III	III
10	III	III	III	III	III

As shown in Table 5, after using the optimization algorithm, the accuracy rate of the rockburst grade evaluation results of PNN has been significantly improved. The evaluation effect of SSA-PNN is the best, with an accuracy rate reaching 90%. The accuracy rates of PSO-PNN and GA-PNN are slightly lower, approximately 80%, and the accuracy rate of PNN is the lowest, at 70%. Therefore, it is reasonable to use SSA-PNN for rockburst grade evaluation in this study.

## 5. Sensitivity analysis of rockburst grade evaluation index

In order to carry out sensitivity analysis of *σ*_θmax_, *σ*_max_, *σ*_c_ and *σ*_cm_ in rockburst grade evaluation, these four parameters were arranged and combined as basic elements. SSA-PNN framework was applied to evaluate the rockburst grade of the test set with the smooth factor unchanged. The results were shown in Table 6.

**Table 6:** Rockburst grade evaluation results various at different combinations.

Combination Number	Evaluation parameters	Accuracy	Precision	Recall rate	F1-score
1	*σ*_max_; *σ*_θmax_; *σ*_c_; *σ*_cm_	0.9000	0.9167	1.0000	0.9545
2	*σ*_max_; *σ*_θmax_; *σ*_c_	0.8000	0.8000	0.9000	0.8445
3	*σ*_max_; *σ*_θmax_; *σ*_cm_	0.9000	0.8584	1.0000	0.9545
4	*σ*_max_; *σ*_c_; *σ*_cm_	0.9000	0.8584	1.0000	0.9545
5	*σ*_θmax_; *σ*_c_; *σ*_cm_	0.8000	0.8000	0.9000	0.8445
6	*σ*_max_; *σ*_θmax_	0.7000	0.8125	0.7500	0.7180
7	*σ*_max_;*σ*_c_	0.8000	0.8000	0.9000	0.8445
8	*σ*_max_; *σ*_cm_	0.8000	0.8572	0.8750	0.8452
9	*σ*_θmax_; *σ*_c_	0.8000	0.8572	0.8750	0.8452
10	*σ*_θmax_; *σ*_cm_	0.8000	0.8000	0.9000	0.8445
11	*σ*_c_; *σ*_cm_	0.4000	0.5417	0.3393	0.4156
12	*σ*_max_	0.5000	0.5000	0.3333	0.6667
13	*σ*_θmax_	0.6000	0.7778	0.6250	0.5572
14	*σ*_c_	0.3000	0.2143	0.3000	0.5000
15	*σ*_cm_	0.6000	0.6191	0.7000	0.6137

As shown in Table 6, the F1-score, precision rate and recall rate of combination 1 are significantly better than those of other combinations; compared with combination 1, the accuracy of combination 2 and combination 5 is slightly lower, and the accuracy of combination 3 and combination 4 is the same as that of combination 1, so when the evaluation parameters of rockburst grade are not easily obtained, combination 3 or combination 4 may be used for rockburst grade evaluation; compared with combination 1, the accuracy of combination 7 ~ 10 (stress-strength factor) is reduced by 0.1, combination 6 (stress factor) by 0.2, and combination 11 (strength factor) by 0.5, indicating that it is reasonable to use stress-strength to evaluate rockburst grade; compared with combination 1, the accuracy of combination 12, 13, 14 and 15 is significantly lower, indicating that the result of rockburst grade by single factor is often different from the actual situation.

Among the four single parameters (*σ*_cm_, *σ*_θmax_, *σ*_max_ and *σ*_c_), *σ*_cm_ and *σ*_θmax_ have the greatest influence on the evaluation, and the accuracy rates of both are 0.6. Keeping *σ*_cm_ and *σ*_θmax_ unchanged, adding *σ*_max_ or *σ*_c_ one by one. It was found that compared with the accuracy rates of combinations *σ*_cm_ and *σ*_θmax_, combinations *σ*_cm_, *σ*_θmax_ and *σ*_max_ increased by 0.1, while combinations *σ*_cm_, *σ*_θmax_ and *σ*_c_ remained unchanged, indicating that *σ*_cm_ and *σ*_θmax_ have the greatest influence on rockburst, followed by *σ*_max_, *σ*_c_ has the least influence on rockburst.

## 6. Discussions

**Fig 8:**
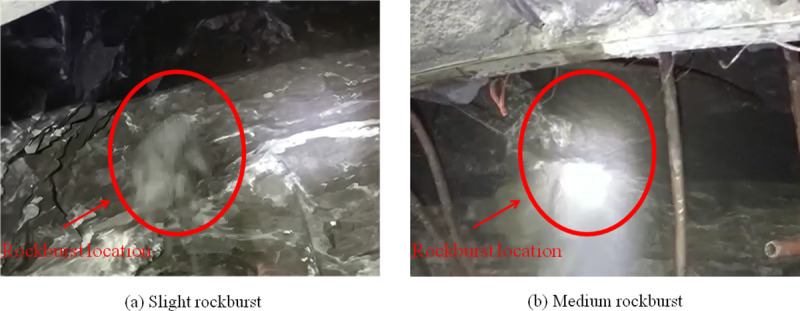
The appearance of rockburst in Mubandling Tunne.

Taking Mubanding tunnel project of Hangzhou-Wenzhou Railway as an example, the applicability of rockburst grade evaluation results by SSA-PNN framework was verified. The Mubandiling Tunnel of Hangzhou-Wenzhou Railway is located in Hangzhou City, Zhejiang Province, China, with a total length of 10240.34 m and a maximum buried depth of 619 m. During the construction, 421 rockbursts occurred in the tunnel, which were concentrated in the vault of the tunnel, and a few rockbursts occurred in the side wall and invert. Fig 8 shows the appearance of rockburst in Mubandling Tunnel.

The main rockburst grade in Mufeiing tunnel is slight and medium rockburst, the duration of rockburst is 2 ~ 8 s, the phenomenon of rock flake spalling and slight ejection can be seen in the light part of rockburst, and the phenomenon of rock ejection, falling and protruding can occur in the serious part of rockburst, and the initial speed of flying rock is large and the direction is not obvious. Rockburst has caused serious damage to Mufeiling tunnel, increased the difficulty of initial support, increased the amount of works and hindered the construction progress. Therefore, it is necessary to evaluate the rockburst grade of Mufeiing tunnel in order to take more appropriate preventive measures. According to the site construction needs, the tests were carried out at DK77 + 500 and DK79 + 050 locations of Mufeiling tunnel. The rockburst grade evaluation was then performed by using SSA-PNN framework, and the results are shown in Table 7.

**Table 7:** Rockburst grade evaluation results in Mufeiling tunnel.

Number	Locations	Actual Grades	Evaluate results	*σ*_θmax_ /MPa	*σ*_max_ /MPa	*σ*_ci_ /MPa	*σ*_cm_ /MPa
1	DK77 + 500	Medium	Medium	66.0	49.7	101.27	3.72
2	DK79 + 050	Medium	Medium	56.1	23.5	35.63	2.12

As can be seen from Table 7, the rockburst grade evaluation results are basically consistent with the actual situation, and the SSA-PNN framework built in this study was well applied in Mufeiling Tunnel.

## 7. Conclusions

In this study, the Lalin Railway tunnel project was taken as an example, and 4 influencing parameters (maximum tangential stress, maximum in-situ stress, uniaxial compressive strength of rock and uniaxial compressive strength of rock mass) of 22 rockburst areas were obtained through indoor and outdoor experiments. These four parameters were then used to form a rockburst grade evaluation index system. A rockburst grade evaluation network was constructed by using SSA (Sparrow search algorithm) and probabilistic neural network (PNN), and the sensitivity analysis of key parameters affecting the evaluation results was also carried out. It shows that:

(1) The proposed SSA-PNN framework demonstrates robust applicability and high precision in rockburst grade evaluation, representing an innovative integration of intelligent optimization with probabilistic learning. This hybrid model effectively overcomes the limitations of conventional deterministic methods by adaptively optimizing the network parameters, thereby enhancing both the accuracy and generalization capability of the rockburst evaluation model.
(2) Sensitivity analysis reveals a novel insight: the uniaxial compressive strength of the rock mass and the maximum tangential stress of tunnel peripheries are the most critical determinants of rockburst grade evaluation accuracy. These findings underscore the necessity of coupling geological strength parameters with stress field characteristics. In contrast, the uniaxial compressive strength of intact rock exhibits minimal impact, which challenges traditional single-parameter evaluation paradigms.
(3) A comparative evaluation shows that using either rock mass strength or stress conditions in isolation leads to an underestimation of rockburst risk. In contrast, the joint consideration of both mechanical strength and in-situ stress conditions yields significantly higher and more realistic rockburst grade assessments. This highlights a key methodological advancement in integrating multi-dimensional geomechanical indicators for enhanced prediction fidelity.
(4) The comprehensive rockburst evaluation strategy established in this study based on the maximum tangential stress, maximum in-situ stress, uniaxial compressive strength of intact rock, and that of rock mass demonstrates high predictive reliability. This multi-factor approach, supported by the SSA-PNN framework, introduces a systematic and data-driven paradigm shift from empirical to intelligent assessment, ensuring greater robustness in practical tunnel engineering applications.

In the rockburst grade evaluation method based on the SSA-PNN framework proposed in this study, the influence of rock mass strength on rockburst was considered, which has certain reference value for tunnel engineering construction and rockburst prevention and control measures. However, at present, the rock mass strength cannot be directly obtained through test methods. The empirical formulas were usually used for estimation, and there may be errors between the estimation results and the actual values. Furthermore, the number of different rockburst grades in the training set of the SSA-PNN network was relatively small, which will have a certain impact on the applicability of the network.

In the subsequent research, the categories and quantities of actual rockburst grades should be increased, the application scope of the proposed neural network evaluation method for rockburst grades need be analyzed, and the influences of factors such as temperature, earthquakes and confined water could be also considered.
